# Clinical characteristics and outcomes of COVID-19 patients in Bali, Indonesia

**DOI:** 10.1371/journal.pone.0269026

**Published:** 2022-06-10

**Authors:** Sri Masyeni, Erni Juwita Nelwan, Rois Muqsith Fatawy, Surya Wibawa, Putu Arya Nugraha, Jarwa Antara, Adi Suparta, D. G. Wedha Asmara, L. G. Sri Yenny, A. A. G. Budhitresna, Dewi Arimas, Dewi Indriani, Kmg Parwata, Ketut Sutarjana, Eka Sugiartha, Siska Kahari, Clareza Arief Wardhana, A. A. G. Indraningrat, Kadek Mulyantari, Arya Widiyana Pasek, Oka Putrawan, Nyoman Trisna Yustiani, Gede Wardana, Made Indra Wijaya, Suka Aryana, Yuli Gayatri, Dewi Dian Sukmawati, Ketut Suastika, Tuti Parwati Merati, Made Bakta, Raka Widiana

**Affiliations:** 1 Department of Internal Medicine, Faculty of Medicine and Health Sciences, Universitas Warmadewa/Sanjiwani Hospital, Bali, Indonesia; 2 Infectious Disease and Immunology Research Center, Indonesia Medical Education and Research Institute, Faculty of Medicine, Universitas Indonesia, Jakarta, Indonesia; 3 Division of Tropical and Infectious Disease, Department of Internal Medicine, Faculty of Medicine, Universitas Indonesia, Jakarta, Indonesia; 4 Bali Mandara Hospital, Bali, Indonesia; 5 Department of Internal Medicine, Pratama Giri Emas Hospital, Bali, Indonesia; 6 Department of Internal Medicine, Nyitdah Tabanan Hospital, Bali, Indonesia; 7 Mangusada Badung Hospital, Bali, Indonesia; 8 Department of Clinical Pathology, Singaraja Hospital, Bali, Indonesia; 9 Klungkung Semarapura Hospital, Bali, Indonesia; 10 Bangli Hospital, Bali, Indonesia; 11 Department of Clinical Pathology, Faculty of Medicine, Universitas Udayana, Bali, Indonesia; 12 Balai Pelatihan Kesehatan Bali, Bali, Indonesia; 13 Department of Internal Medicine, Sanglah Hospital, Faculty of Medicine, Universitas Udayana, Bali, Indonesia; Nagasaki University: Nagasaki Daigaku, JAPAN

## Abstract

**Introduction:**

The spectrum of illness and outcomes of coronavirus disease 2019 (COVID-19) patients may vary. This study reports the characteristics of COVID-19 patients in Bali, Indonesia, and evaluates the diagnostic value of their clinical symptoms.

**Method:**

This observational study was conducted in eight hospitals. The patients were classified as non-severe COVID-19, severe COVID-19, and non-COVID-19. Demographics, clinical, laboratory, and radiologic characteristics, and outcomes of COVID-19 patients were collected. Factors associated with the severity and outcomes were assessed using the chi-squared test or ANOVA when appropriate. We also compared the clinical features of non-severe COVID-19 and non-COVID-19 patients to evaluate the diagnostic accuracy.

**Results:**

This study included 92 patients: 41 non-COVID-19 and 51 COVID-19 patients, comprising 45 non-severe and six severe cases. The most common symptoms of COVID-19 were cough (47.1%), fever (31.0%), and dyspnea (25.3%). Cough, fatigue, and anosmia have high accuracy, and combining these complaints in clinical diagnostics offered a higher accuracy in predicting COVID-19 patients (60.1%). We found lower lymphocyte counts and interleukin-1R levels and higher levels of C-reactive protein, interleukin-6, and interleukin-8 in severe compared than in non-severe COVID-19 patients. Lactate dehydrogenase was associated with intensive care unit admission and ventilator use, while other markers such as neutrophil-lymphocyte ratio, C-reactive protein, and interleukin-6 were not.

**Conclusion:**

A battery of symptoms, including cough, fatigue, and anosmia, is likely associated with COVID-19 in Bali. Clinicians should be aware of these symptoms to ensure a prompt diagnostic test for COVID-19, beyond other causes of acute febrile illnesses.

## Introduction

The current coronavirus disease 2019 (COVID-19) pandemic, caused by the severe acute respiratory syndrome coronavirus 2 (SARS-CoV-2), is a global threat. This virus is closely linked to previous coronaviruses that caused severe acute respiratory syndrome (SARS) and Middle East respiratory syndrome (MERS) outbreaks in 2002 and 2012, respectively [1]. As of December 27, 2021, more than 296 million COVID-19 cases and more than 5.5 million deaths have been reported globally [2]. The clinical spectrum of COVID-19 ranges from asymptomatic to mild, moderate, and severe, which may lead to death [3]. Most people infected with SARS-CoV-2 are asymptomatic or complain of pneumonia-like symptoms, including fever, cough, shortness of breath, and viral symptoms, such as myalgia, fatigue, and headache [4]. Older patients or those with comorbidities are likely to have more severe symptoms [1, 4–6]. Asymptomatic COVID-19 patients typically share challenges for disease management. This group can potentially spread the virus, which is difficult to trace. Conversely, symptomatic patients may mimic other acute febrile illnesses or diseases due to pneumonia, such as tuberculosis, influenza, or streptococcal pneumonia [7, 8].

Indonesia, a tropical country, has been infected with COVID-19 since March 2020, which has increased rapidly to date [9]. COVID-19 cases have been reported in all areas of Indonesia, including Bali [9]. The COVID-19 pandemic has had massive detrimental effects on the economy, as tourism is the main source of income. No data have described the exact clinical differences between COVID-19 and non-COVID-19 patients in Bali. This study aimed to compare the disparity in clinical and laboratory manifestations disparity between symptomatic COVID-19 and non-COVID-19 patients in Bali. Our study’s diagnostic value may help clinicians differentiate between key signs and symptoms between these groups of patients.

## Materials and methods

### Study design and population

This observational study evaluated the clinical and epidemiological characteristics of patients with COVID-19 symptoms in Bali, Indonesia. The minimum sample size of 49. The sample size assumption was based on a 15% COVID-19 positive rate for the trial, with the alpha set at 5% and an acceptable prediction error set at 10%. The total sample of 92 consisted of participants who fulfilled the following criteria: (1) patients with signs and symptoms of COVID-19 within the first week of illness, (2) 18 years of age or older, and (3) ability to provide informed consent.

### Study settings

**Fig 1:**
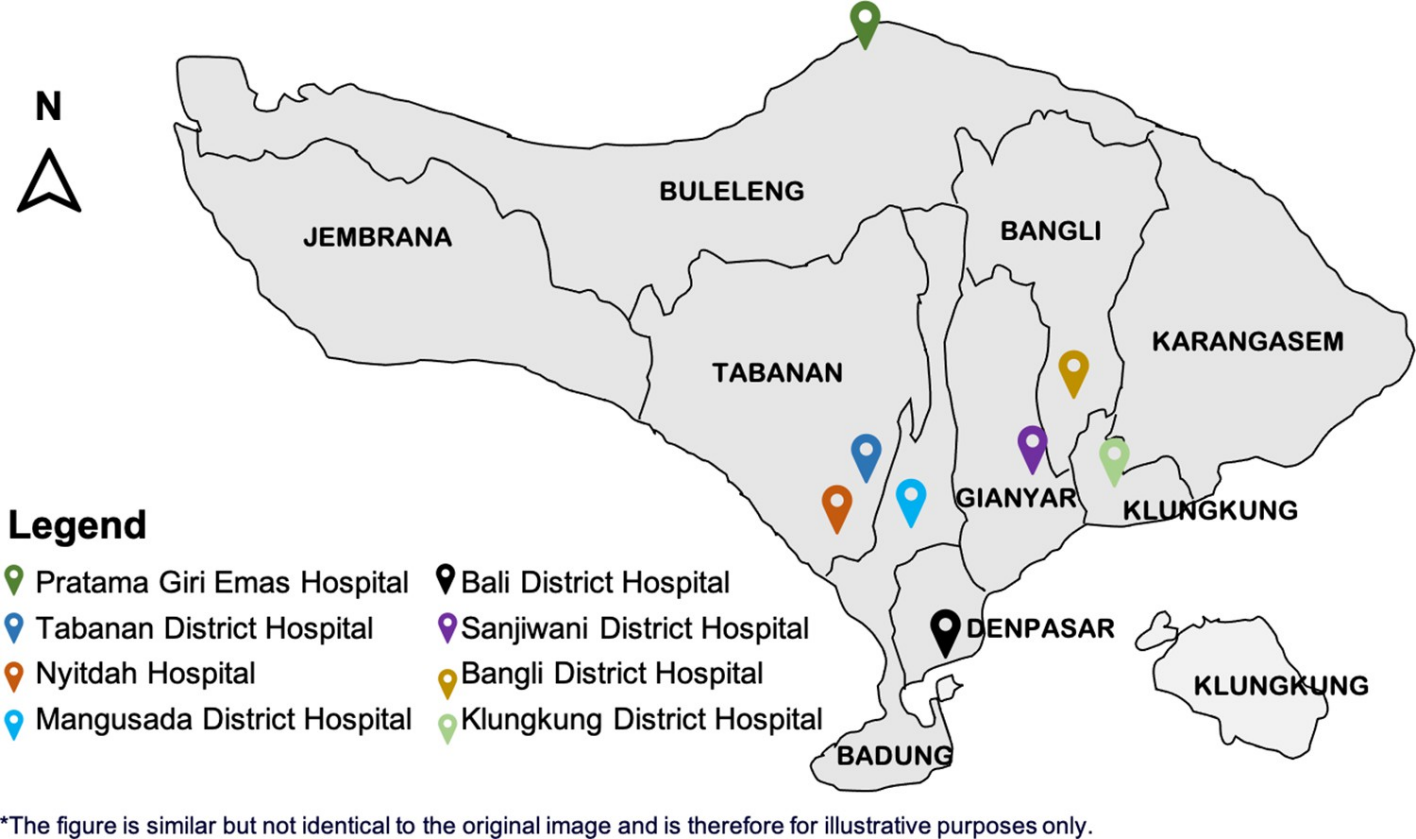
Plotted map of hospitals in Bali where patients recruited.

This study was conducted in many hospitals involved with the Indonesian Society of Internal Medicine. Patients were recruited from eight hospitals: Bangli District Hospital, Klungkung District Hospital, Sanjiwani District Hospital, Bali Mandara Hospital, Mangusada District Hospital, Pratama Giri Emas Hospital, Nyitdah Hospital, and Tabanan District Hospital, from May to August 2020 (Fig 1). Adult patients (≥18 years old) admitted with COVID-19 symptoms at the study sites, including fever, cough, dyspnea, and anosmia, were enrolled in the study using consecutive sampling techniques. Patients who were asymptomatic or refused to sign an informed consent form were excluded from the study. COVID-19 patients were categorized into two groups: non-severe COVID-19 and severe COVID-19. The non-severe COVID-19 group consisted of mild and moderate COVID-19 patients, and the severe COVID-19 group consisted of severe and critically ill COVID-19 patients, according to the NIH criteria [10]. For comparison, those with COVID-19-like symptoms with negative real-time polymerase chain reaction (RT-PCR) results were categorized as the non-COVID-19 group (mainly diagnosed as non-COVID-19 pneumonia, dengue hemorrhagic fever, and upper respiratory tract infections).

### Ethical consideration

This study was approved by the Ethical Board of the Faculty of Medicine, Udayana University, Bali (No. 941/UN14.2.2.VII.14/LT/2020), and written informed consent was obtained from all the patients prior to enrolment.

### Severity classification

COVID-19 patients were classified as having non-severe or severe illnesses. Non-severe COVID-19 consisted of mild and moderate symptomatic patients, whereas the severe group comprised patients with severe and critical illness. Mild illness confirmed positive individuals with signs and symptoms of COVID-19, but who did not have shortness of breath, dyspnea, or abnormal chest imaging. Moderate illness was defined as abnormal chest imaging and saturation of oxygen (SpO2) ≥94% with room air at sea level [10]. Individuals with severe illness have SpO2 <94%, respiratory frequency >30 breaths/min, or lung infiltrates >50%, whereas critical individuals have respiratory failure, septic shock, and/or multiple organ dysfunction [10].

### Clinical and outcome variables

Demographic data and clinical characteristics were obtained by interviewing patients. Laboratory parameters, comorbidities, and patient outcomes were assessed. Admission to the intensive are unit (ICU), need for a ventilator, and death were recorded as primary outcomes.

### Laboratory examinations

Laboratory tests were performed to measure the levels of hemoglobin, lymphocyte and platelet counts, neutrophil-lymphocyte ratio (NLR), lactate dehydrogenase (LDH), C-reactive protein (CRP), TNF-α, interleukin (IL)-1R1, IL-6, IL-8, and IL-10. The serum LDH level was measured using a Cobas c50, an enzymatic method based on the ultraviolet (UV) assay principle [11], while a particle-enhanced immunoturbidimetric assay was used to measure the serum CRP level [12]. The levels of TNF-α, IL-1R1, IL-6, IL-8, and IL-10 were measured by enzyme-linked immunosorbent assay using reagents from Elabscience^®^ [13–17]. Nasopharyngeal swabs from all patients were obtained and examined for SARS-CoV-2 using the Liferiver Novel Coronavirus (2019-nCoV) Real-Time Multiplex RT-PCR Kit (BioVendor, Czech Republic).

### Clinical diagnostic value

The diagnostic value of our study was adopted from a previous study assessing enteric fever [18]. Briefly, every symptom and sign that appeared in mild-moderate COVID-19 and non-COVID-19 patients were evaluated. We then categorized the symptoms into true positive (positive symptom with positive reverse transcription polymerase chain reaction [RT-PCR] results), false positive (positive symptom with negative RT-PCR), false negative (negative symptom with positive RT-PCR results), and true negative (negative symptom with negative RT-PCR results). Sensitivity and specificity were calculated to determine the overall accuracy. The receiver-operating characteristic curve (ROC) was plotted with sensitivity as the Y-axis and 1-specificity as the X-axis for each symptom and sign. Signs and symptoms with an accuracy of more than 50% and lying above the 45-degree line of the ROC were considered significant. Symptoms accounting for more than 60% accuracy and plotted lying above the 45° line of ROC were categorized as major symptoms, whereas symptoms ranging between 50%-59% accuracy and plotted lying above the 45° line of ROC were recognized as minor [19]. Diagnostic criteria were then proposed based on major and minor symptoms to make up the proposed clinical diagnostic criteria.

### Statistical analysis

Factors associated with severity and outcomes among the groups were assessed using the chi-squared test or ANOVA when appropriate. Determinants that were significantly associated among the groups during chi-squared or ANOVA were further assessed using post-hoc analysis, and the main purpose was to determine the determinants associated with severe COVID-19 compared to mild-moderate COVID-19 and non-COVID-19. In addition, factors associated with outcome and death were assessed using logistic regression with a predictive model to control for confounding variables. Statistical analyses were conducted using Statistical Package of Social Sciences software (version 21.0; SPSS Inc., Chicago, IL, USA).

## Results

### Demographics and clinical characteristics

At the end of the study, 92 patients with COVID-19-like symptoms were enrolled; 51 of them were confirmed COVID-19 positive using RT-PCR. Of the COVID-19 patients, 45 (48.9%) were classified as having non-severe COVID-19 and 6 (6.5%) as having severe COVID-19. Non-severe COVID-19 patients comprised 26 (28.3%) mild cases and 19 (21.1%) moderate cases. Additionally, COVID-19-like illness or non-COVID-19 patients (41; 45.6%) were included in this study, which mostly consists of pneumonia non-COVID-19 (24; 26.1%), dengue hemorrhagic fever (5; 5.4%), and upper respiratory tract infection (5; 5.4%).

The mean age of patients was 50.8±15.7 years; the majority were men (58.7%), and 41.3% had at least one comorbidity such as hypertension and diabetes (**Table 1**). The most common complaint on hospital admission was fever (42.4%), whereas the most common signs and symptoms were cough (67.4%), fever (59.8%), and dyspnea (44.6%). The demographic and clinical characteristics of patients are presented in **Table 1**.

**Table 1:** Demographics and baseline characteristics of patients infected with COVID-19 and non-COVID-19 patients.

	All patients (N = 92)	Non-COVID-19 patients (N = 41)	Non-severe COVID-19 patients (N = 45)	Severe COVID-19 patients (N = 6)	*p* value
**Characteristics**					
Age, mean (SD)	50.8±15.7	54.01±17.2	49.7±14.86	55.47±8.44	0.289*
Sex					
Men	53 (57.6%)	23 (25%)	25 (27.2%)	5 (5.4%)	0.509**
Women	39 (42.4%)	18 (19.6%)	20 (21.7%)	1 (1.1%)	0.509**
Any comorbidity					
Hypertension	21 (22.8%)	7 (17.1%)	11 (24.4%)	3 (50%)	0.161**
Diabetes	11 (12%)	3 (7.3%)	6 (13.3%)	2 (33.3%)	0.144**
Pulmonary diseases	6 (6.5%)	3 (7.3%)	1 (2.2%)	1 (16.7%)	0.161**
Malignancy	3 (3.3%)	2 (4.9%)	1 (2.2%)	0	0.677**
Heart diseases	6 (6.5%)	3 (7.3%)	3 (6.7%)	0	1.000**
Multi comorbidity	9 (9.8%)				
Sign and symptoms					
Fever	55 (59.8%)	28 (68.3%)	21 (46.7%)	6 (100%)	0.012**
Sore throat	10 (10.9%)	2 (4.9%)	6 (13.3%)	2 (33.3%)	0.079**
Runny nose	6 (6.5%)	1 (2.4%)	2 (44.4%)	3 (50%)	0.003**
Cough	62 (67.4%)	21 (51.2%)	35 (77.8%)	6 (100%)	0.006**
Dyspnea	41 (44.6%)	19 (46.3%)	16 (35.6%)	6 (100%)	0.008**
Myalgia	20 (21.7%)	7 (17.1%)	9 (20%)	4 (66.6%)	0.041**
Anosmia	10 (10.9%)	1 (2.4%)	8 (17.8%)	1 (16.7%)	0.042**
Fatigue	28 (30.4%)	7 (17.1%)	18 (40%)	3 (50%)	0.029**
Nausea	14 (15.2%)	3 (7.3%)	5 (11.1%)	6 (100%)	<0.001**

Data are presented as mean±SD deviation or N(%), where N is the total number of patients. *p* values comparing non-CoV, non-severe CoV, and severe CoV patients are from the X^2^ test, Advance-Fisher test** test, and one-way ANOVA *.

Patients with severe symptoms were older (55.47±8.44 years) vs. patients with mild-moderate illness (49.7±14.86 years) (**Table 1**). All participants of the severe group had at least one comorbidity present, spanning across hypertension, diabetes, and/or pulmonary disease. Multiple comorbidities such as diabetes mellitus-hypertension reported in 3 patients (3.3%), while diabetes mellitus and congestive heart failure (CHF), chronic obstructive pulmonary disease (COPD) and tuberculosis (TBC), and diabetes mellitus with malignancy were found in 3 (3.3%), 1 (1.1%), and 1 (1.1%) cases, respectively. Moreover, all patients in the severe group experienced fever, cough, dyspnea, and nausea. Age, history of pulmonary disease, and the manifestation of fever, cough, dyspnea, myalgia, and nausea were significantly different among the three groups (non-COVID-19, non-severe COVID-19, and severe COVID-19).

### Laboratory and chest X-ray characteristics

Laboratory findings of the study participants at the time of admission are presented in **Table 2**. There were significant differences between the non-COVID-19, mild-moderate-COVID-19, and severe-COVID-19 samples in terms of lymphocyte count, NLR, LDH, CRP, IL-6, and IL-8.

**Table 2:** Comparison of laboratory finding of all samples involved in the study.

Variables	All patients (N = 92)	Non-Covid-19 patients (N = 41)	COVID-19		*p* value
			Non-severe Covid-19 patients (N = 45)	Severe Covid-19 patients (N = 6)	
White blood cell count, x 10^3^/L^b^	8.2 (1.5–30.4)	8.43 (1.5–30.4)	7.78 (3.89–23.2)	11.3 (5.67–13)	0.299**
Lymphocyte count,	1.95±0.9	1.31±0.61	1.79±0.89	0.75±0.26	0.001**
x 10^3^/L^a^					
NLR^b^	2.5 (0.6–23.7)	5.2 (1.26–23.7)	3 (0.6–23.5)	12.9 (3.5–22.9)	0.001**
Haemoglobin, g/L^a^	13.4±1.73	12.67±2.05	13.08±1.54	13.19±0.8	<0.837**
Platelet count,	252 (33–782)	255 (33–519)	257 (70–782)	169 (136–340)	0.181**
x 10^3^/L^b^					
Lactate dehydrogenase U/L^b^	332 (180–979)	398 (193–916)	327 (199–719)	924,5 (406–979)	0.001**
C-reactive protein^b^	2.0 (0.3–385.8)	22.14 (0.3–363.85)	2.09 (0.3–305.1)	114.35 (26.99–385.81)	<0.001**
TNF-α,pg/mL^b^	2.1 (0.1–92.6)	2.03 (0.14–92.41)	1.32 (0.26–78.36)	2.1 (1.01–4.64)	0.611**
IL-6, pg/mL^b^	6.9 (3.5–1350.9)	11.72 (3.69–727.27)	5.35 (3.51–299.51)	108.5 (9.49–1350.85)	<0.001**
IL-8 pg/mL^b^	9.7 (1.1–87.4)	17 (6.14–87.39)	9.19 (2.23–69.39)	19.34 (7.81–79.61)	<0.001**
IL-10 pg/mL^a^	11.2 (0.83–40)	11.25±6.94	10.98±5.72	8.35±2.61	0.564*
IL-1R, pg/mL^b^	0.04 (0.01–0.4)	0.03 (0.01–0.4)	0.03 (0.01–0.2)	0.03 (0.01–0.05)	0.305**

Data are ^a^mean±SD, ^b^median (IQR), or n/N (%), where N is the total number of patients. *p* values comparing Non-Covid-19, mild Covid-19 and moderate-severe Covid-19 patients are from ANOVA* and Kruskal-Wallis** tests. NLR is neutrophil-to-lymphocyte ratio

Post-hoc tests were conducted to further investigate the differences of significant laboratory findings between those groups (**Table 3**). Lymphocyte count in severe disease patients was significantly lower than the non-severe group (p<0.001) and non-COVID-19 patients (p = 0.02). However, lymphocyte count in non-severe COVID-19 patients was significantly higher than non-COVID-19 patients (p = 0.009).

**Table 3:** A post-hoc test of the significant laboratory findings.

Variables	p-value of the post-hoc test		
	Severe COVID-19 *vs*. Non-COVID-19 patients	Severe COVID-19 *vs*. Non-severe COVID-19	Non-severe COVID-19 *vs*. Non-COVID-19
Lymphocyte count	0.020	<0.001	0.009
Neutrophil lymphocyte ratio	0.018	0.001	0.012
Lactate dehydrogenase	<0.001	<0.001	0.233
CRP	0.029	0.001	<0.001
IL-6	0.056	0.001	0.002
IL-8	0.503	0.016	<0.001

The NLR was significantly higher in severe patients compared without non-COVID-19 (p = 0.018) and mild-moderate patients (p = 0.001) (**Table 3**). Nevertheless, the NLR in the COVID-like group was higher relative to the mild to moderate group (p = 0.012). The levels of LDH were significantly higher in severe COVID-19 patients than in non-COVID-19 patients and non-severe COVID-19 patients (p<0.001 for each comparison). CRP levels in severe COVID-19 patients were also significantly higher than those in non-COVID-19 (p = 0.029) and non-severe COVID-19 patients (p<0.001).

The levels of IL-6 were significantly higher in severe disease than in mild-to-moderate conditions (p<0.001) (**Table 3**). Patients with severe COVID-19 had considerably higher levels of IL-8 than non-severe patients (p = 0.016). However, the non-COVID group had a higher level of IL-8 than the mild to mild-moderate disease group. Most chest X-rays showed pneumonia in at least 39.8% of symptomatic cases, of which 29.4% revealed bilateral infiltrates. Signs of bronchitis were found in 3.1% of cases and pleural effusion was found in 1.6% of symptomatic cases.

### Clinical outcomes

The details of the clinical outcomes of the study participants are presented in **Table 4**. Of the 92 patients, 26 (19.4%) required ICU admission, nine (9.7%) were on a ventilator, and seven (7.7%) died (**Table 4)**. Of the total COVID-19 cases, 23.5% required intensive care in the ICU and 11.7% required ventilators.

**Table 4:** Outcome comparison of COVID-19 and non-COVID-19 patients.

Variables	All patients (N = 92)	Non-Covid-19 patients (N = 41)	Covid-19 patients (N = 51)	*p* value
ICU treatment	26 (19.4%)	14 (34.1%)	12 (23.5%)	0.381
On ventilator	9 (9.7%)	3 (7.3%)	6 (11.7%)	0.291
Death	7 (7.7%)	5 (12.1%)	2 (3.9%)	0.320

N(%), where N is the total number of patients. *p* values comparing Non-Covid-19, and Covid-19 patients are from the X^2^ test and Fisher exact test.

Logistic regression was conducted to assess the most significant blood marker(s) associated with the COVID-19 outcomes (**Table 5**). Our study found that LDH was significantly associated with ICU admission (p = 0.037) and ventilator usage (p = 0.016). No blood markers were associated with mortality associated with COVID-19.

**Table 5:** Logistic regression for factors associated with COVID-19 outcomes.

Variables	Outcomes					
	ICU admission		On ventilator		Death	
	Exp(B)	p-value	Exp(B)	p-value	Exp(B)	p-value
Lactate dehydrogenase	1.00	0.037*	1.01	0.016*	0.99	0.724
C-reactive protein	1.01	0.106	1.00	0.766	1.00	0.786
TNF-α	1.01	0.268	0.89	0.648	0.74	0.409
IL-6	0.99	0.744	1.00	0.504	1.00	0.923
IL-8	0.99	0.663	0.98	0.525	1.01	0.777
IL-10	0.92	0.112	1.06	0.431	0.88	0.218
IL-1R	21.91	0.446	0.00	0.422	0.00	0.315

* Significant at p = 0.05

Our data suggested that severe COVID-19 patients with ventilators increased the mortality up to 26-fold compared to those who did not require ventilator (OR: 26.16, 95%CI: 3.13–218.60, p = 0.003). In addition, ICU admission did not associate with death (OR: 2.52; 95%CI: 0.25–25.53, p = 0.432).

### Diagnostic value

**Fig 2:**
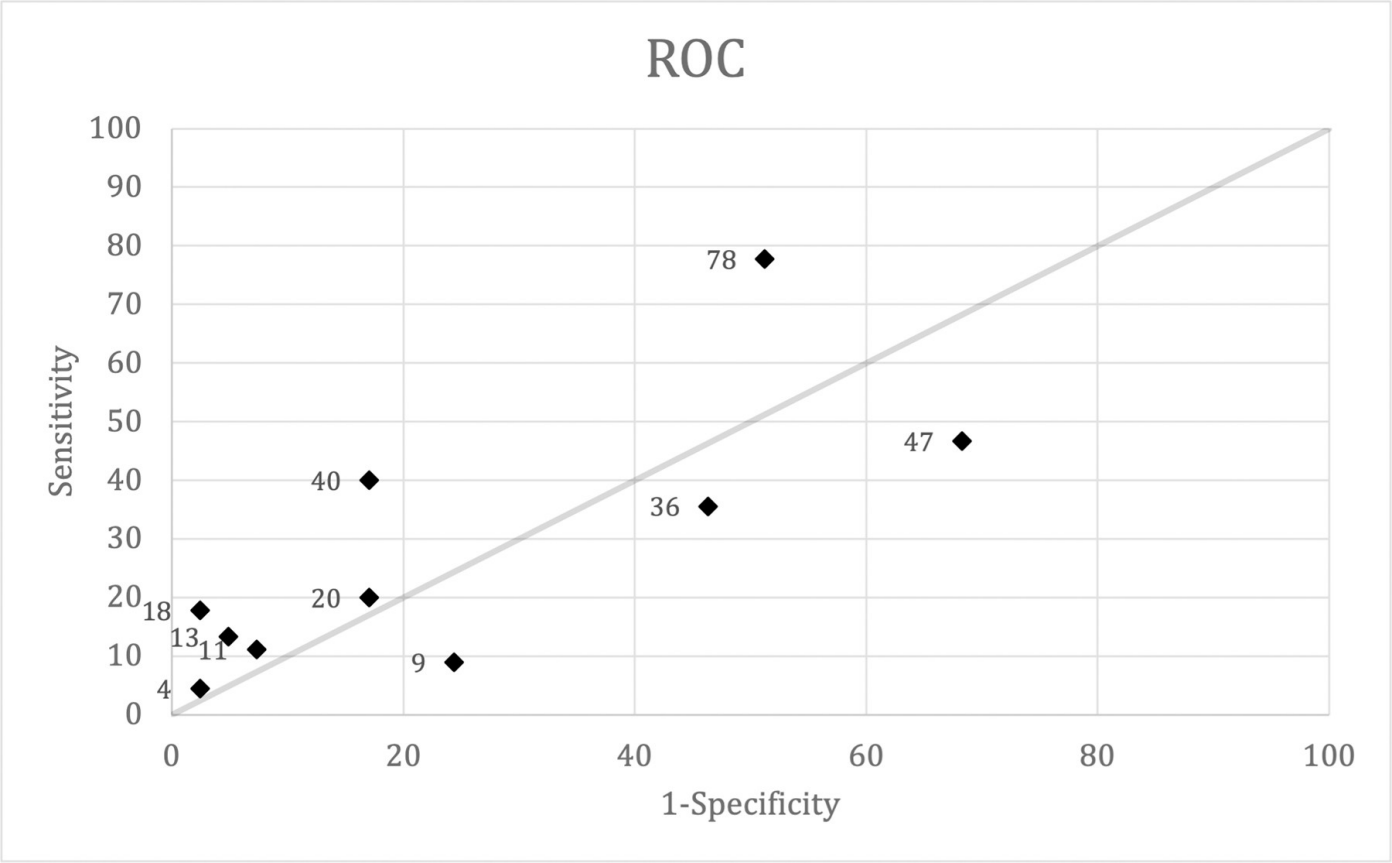
Receiver operating characteristics curve. Plotting clinical characteristics.

At least six symptoms, including cough, fatigue, anosmia, sore throat, myalgia, and nausea, had an overall accuracy equal to or greater than 50% **(Table 6)**. Cough and fatigue were the major symptoms, whereas the other four accounted for minor symptoms in the non-severe COVID group. Cough was the highest in terms of sensitivity (77.78%), positive-negative predictive value (62.50%-66.67%), and accuracy (63.95%). Although fever was the second most common complaint in COVID-19 patients in this study, it had low specificity (31.71%) and accuracy (39.53%). ROC curves were obtained by plotting sensitivity and 1-specificity **(Table 7)**. The curve revealed that seven out of ten plots of clinical characteristics were considered significant symptoms (Fig 2). The accuracy of the diagnostic criteria proposed by incorporating cough, fatigue, anosmia, and sore throat ranged from 54.30% to 58.11%. However, criteria A (C-19), combining cough, anosmia, and fatigue, offered more accuracy (59.39%) while maintaining its specificity and positive predictive value **(Table 8)**.

**Table 6:** Diagnostic accuracy of symptoms and signs.

Symptoms and Laboratory Features	sensitivity (a/a+c %)	specificity (d/d+b %)	Positive predictive value (a/a+b %)	Negative predictive value (d/d+c %)	Overall Accuracy (a+d/a+b+c+d) %
Fever	46.67	31.71	42.86	35.14	39.53
Sore throat	13.33	95.12	75.00	50.00	52.33
Runny nose	4.44	97.56	66.67	48.19	48.84
Cough	77.78	48.78	62.50	66.67	63.95
Dyspnea	35.56	53.66	45.71	43.14	44.19
Myalgia	20.00	82.93	56.25	48.57	50.00
Anosmia	17.78	97.56	88.89	51.95	55.81
Fatigue	40.00	82.93	72.00	55.74	60.47
Nausea	11.11	92.68	62.50	48.72	50.00
Thrombocytopenia	8.89	75.61	28.57	43.06	40.70

**Table 7:** True positive and false negative rate.

Symptoms and/or laboratory features	Sensitivity (True positive rate) %	1-Specificity (False positive rate) %
Fever	47	68
Sore throat	13	5
Runny nose	4	2
Cough	78	51
Dyspnea	36	46
Myalgia	20	17
Anosmia	18	2
Fatigue	40	17
Nausea	11	7
Thrombocytopenia	9	24

**Table 8:** Clinical diagnostic accuracy for each criterion.

Proposed Criteria for Clinical Diagnostic	sensitivity a/a+c %	specificity d/d+b %	Positive predictive value a/a+b %	Negative predictive value d/d+c %	Overall Accuracy a+d/a+b+c+d in %
Criteria A or C-19: Cough, Anosmia, and Fatigue	45.19	76.42	67.78	55.95	60.08
Criteria B: 2 major 2 minor	36.89	79.15	66.05	53.32	57.03
Criteria C: 2 major 3 minor	33.30	70.57	56.55	50.46	51.20
Criteria D: 1 major 4 minor	38.46	75.38	63.46	53.24	56.11
Criteria E: symptoms and/or laboratory features with plot lying above 45° line	30.00	83.33	66.39	52.03	55.43

## Discussion

Clinical and epidemiological characteristics of COVID-19 patients from eight tertiary hospitals and one laboratory in Bali, Indonesia were analyzed in this study. From 51 positive COVID-19 patients, we observed more men than women among the 51 positive COVID-19 patients, similar to previous studies [3, 6, 20]. More male-to-female patients were also observed during SARS and MERS outbreaks in 2002 and 2012 [21, 22]. Female sex hormones and the X chromosome, which play essential roles in innate and adaptive immunity, are suggested to protect females from viral infections, including SARS-CoV-2 [23]. However, another study with a larger population showed that most COVID-19 patients were women [5]. Differences in the proportion of males to females among COVID-19 patients in a particular area might be due to different exposures to SARS-CoV-2 or the practice of preventive measurements.

This study found that the average age of those with non-severe COVID-19 and severe COVID-19 were 43.5±16.5 and 45.5±8.4 years and all patients with severe COVID-19 symptoms had at least one comorbidity present (diabetes, hypertension, or pulmonary diseases). This is consistent with previous studies, which suggested that most patients with COVID-19 were aged 34–59 years old [1, 5, 20, 24]. Reports have also suggested that SARS-CoV-2 is more likely to infect older men with comorbidities due to weaker immune function [21, 22, 25]. The immune status of patients plays an important role in the severity of viral diseases besides the pathogens’ virulence. People with weak immune functions, such as the elderly, people with HIV, diabetes, pregnant women, or immunosuppressive therapy, have a higher risk of being infected with SARS-CoV-2 and higher mortality risk [6]. Previous reports have also shown that older age and comorbidities are associated with more severe disease and poorer outcomes in COVID-19 [4, 5].

Regarding laboratory findings, the lymphocyte count was significantly lower in patients with severe COVID-19 than in COVID-19 patients with mild or non-severe symptoms. This finding supports several studies suggesting that lymphocytopenia is associated with disease severity and poor prognosis of COVID-19 [26, 27]. Although the mechanism of lymphopenia in severe COVID-19 is not fully understood, several possible underlying causes have been proposed: (1) the inflammatory cytokine storm that affects T and Natural Killer cells (NK cells); (2) exhaustion of T cells due to SARS-CoV-2 infection; (3) SARS-CoV-2 infects T cells; and (4) SARS-CoV-2 infection interferes with the expansion of T cells, all of which result in decreased lymphocyte count [27].

The platelet count in severe COVID-19 patients was significantly lower than that in both non-COVID-19 and mild-moderate disease patients, supporting a previous study that suggested reduced production of platelets in damaged lungs since SARS-CoV-2 infection caused thrombocytopenia in COVID-19 patients [26]. Our findings also confirm results from another study which revealed that thrombocytopenia is associated with an increased rate of disseminated intravascular coagulation (DIC), impaired coagulation function, severe disease manifestation, and an increase in mortality rate [28]. Low platelet counts are also linked to high D-dimer levels, which can predict severe and fatal cases [29].

The present study also found that NLR, LDH level, and CRP level were significantly higher in patients with severe COVID-19 than in those with non-severe COVID-19, in agreement with previous studies that suggested NLR, LDH, and CRP level as prognostic factors for COVID-19 severity [20, 27, 30, 31]. Our study also found a higher expression of IL-6 and IL-8 in patients with severe COVID-19 than in those with mild COVID-19. Higher plasma levels of proinflammatory cytokines such as IL-1, IL-2, TNF-α, GM-CSF, and MCP1 have been reported in COVID-19 patients [20]. A previous study also suggested that IL-6 and IL-10 levels were significantly increased in patients with severe COVID-19 and were strong predictors of severe disease [32].

Our data suggest that a high LDH level is significantly associated with ICU admission and ventilator use. A previous study showed that severity increased 6-fold and mortality increased 16-folds in COVID-19 patients with elevated LDH [33], and elevated LDH levels were negatively correlated with survival days (p = 0.022) [34]. Another study showed that the proportion of elevated LDH levels was higher in the non-survivor group than in the survivor group (98% *vs*. 54%, p<0.01) [35]. The lung tissue damage caused by SARS-CoV-2 infection induces LDH release, resulting in a greater amount of LDH in circulation. A meta-analysis also suggested LDH as a severity marker and predictor of survival in COVID-19 [36].

The ROC plot revealed that cough, fatigue, and anosmia were more significant in mild-to-moderate symptomatic COVID-19 and COVID-19-like-illness. In this study, fever and cough appeared most frequently, similar to previous studies [37, 38]. However, fever had considerable sensitivity but lacked specificity and predictive value. Moreover, runny nose, dyspnea, myalgia, and nausea have low sensitivity and predictive value but high specificity.

Shared clinical and laboratory features of COVID-19 and dengue fever pose a serious issue since their similarities may lead to misdiagnosis among clinicians [39]. Several case studies from Singapore, Thailand, and Indonesia have reported misdiagnosed COVID-19 patients with dengue fever after thrombocytopenia and tested positive for dengue Immunoglobulin M (IgM) as well as positive non-structural protein 1 (NS1) and Immunoglobulin G (IgG) in some cases [39–41]. Moreover, our study found that 4/45 (8.7%) symptomatic COVID-19 patients had thrombocytopenia, and 1/45 (2.2%) patients tested positive for the NS1 antibody. This finding corroborated that screening for dengue in blood samples could be positive in COVID-19 patients. Furthermore, NS1 positive in COVID-19 patients may suggest coinfection between both diseases, particularly in areas where dengue is prevalent. However, additional studies are required to confirm this. As Bali is known to be an area where dengue is hyperendemic [42] and its prevalence amidst the COVID-19 pandemic is the second-highest in the country, these findings should raise awareness among public health authorities and health workers. In clinical practice, both diseases should be diagnosed meticulously.

After testing our proposed criteria, C-19, consisting of cough, fatigue, and anosmia, provided the highest accuracy (60.1%) with 45.2% sensitivity and 76.4% specificity (p = 0.0005; x2 = 12.3; df = 1). Cough and fatigue are common symptoms of coronavirus disease (COVID-19). Another study in Mexico suggesting that combination of ≥ 3 symptoms and signs (fever, cough, anosmia, dyspnoea and oxygen saturation <93%, and headache) provided accuracy of 63.2% with 67.39% sensitivity and 58.24% specificity [43]. In this study, anosmia comprised only 8/45 (17.8%) symptomatic patients. Nevertheless, a meta-analysis study revealed that the mean prevalence of smell dysfunction differs between ethnicities, with 44.1% of Caucasians and only 22.4% of the East Asia population suffering [44]. We emphasize that the accuracy of criteria C-19 does not offer a high value; however, it is worth applying as the first COVID-19 screening. Based on these findings, we suggest that clinicians promptly request an RT-PCR test when identifying patients with criteria C-19.

Our study had some limitations. First, a small number of COVID-19 confirmed cases (n = 51) were included in this study. Studies with a larger sample size from all over Bali, even across the country, are required to provide a better diagnostic clue for COVID-19 patients. Second, we did not include D-dimer assays because it is an essential parameter for determining the severity of COVID-19 [29]. Third, because the study was conducted in the early phase of the pandemic, data collection was limited. Fourth, we did not do genotyping analysis so we could not compare the clinical characteristics of each variants. Despite these limitations, this study is the first to explore the clinical characteristics of COVID-19 patients in Bali, which may help enrich the existing literature on clinical characteristics that could help manage COVID-19 patients in countries with limited resources, such as Indonesia.

## Conclusions

Our study highlighted that cough, fatigue, and anosmia were more significant than other clinical features in determining the presence of COVID-19. This study also suggests that older men with comorbidities are at a higher risk of contracting COVID-19. The lymphocyte level was lower, while the levels of NLR, LDH, CRP, IL-6, and IL-8 were higher in patients with severe COVID-19 than in those with non-severe symptoms. LDH was associated with ICU admission and ventilator use. The findings of this study will enrich the current knowledge on COVID-19, particularly in Indonesia, with limited data available. As one of the famous tourist destinations in Indonesia, the Bali government should increase its efforts to strengthen surveillance systems and improve laboratory capacity and healthcare facilities to control the pandemic in order to welcome visitors from all over the world.

## Supporting information

S1 Dataset Study’s minimal underlying dataset. (XLSX)
